# Hospital and Institutional News

**Published:** 1916-01-15

**Authors:** 


### HOSPITAL AND INSTITUTIONAL NEWS.

### HOSPITAL REPAIRS AND MAINTENANCE.

A striking illustration of the value of thoroughness in the internal organisation of the department of a hospital will be found on page 353 of the present issue on the subject of hospital repairs and maintenance. We hope every practical reader will study the article, and we shall hope to learn later its actual monetary value in pounds sterling. It contains information and evidence which is bound to appeal to every man of business who makes it his practice to contribute generously and systematically to the support of hospitals. Our continuous effort is to make The Hospital more and more the helper and friend of each hospital worker everywhere. In such an enterprise the amount of help it is possible to give depends largely on the active interest and co-operation of each worker concerned. We welcome the ever-extending co-operation of the best workers in many departments of the hospital field. The crusade we have waged for years in favour of higher remuneration for the chief officials has produced men of a higher type, education, and qualifications for these posts, and has influenced the ever-extending efficiency of the administration of some of the greatest hospitals in the country. Thanks to this active co-operation on the part of the officials of the principal hospitals we are now in a position to publish evidence of the ever-increasing efficiency of the work done and the economical effects of the thoroughness with which each department is worked. We hope the article on page 353 may induce many workers up and down the country to send us similar instructive details on each of the great departments of a hospital, for the whole or some of which they may be responsible.

### GIFTS IN MEMORY OF THE KILLED IN ACTION.

We are all acquainted with the practice of endowing beds in memory of friends or relatives who have died, but a useful extension of the practice is to be recorded in the donation of smaller sums in memory of those who have fallen at the Front. This motive is being acted on in Scotland, and differs from the practice of endowment in that the donors do not expect to have the name of the person commemorated associated with the institution in the same formal way as is the case when a bed is named or a piece of furniture given bearing an inscription on a metal plate. The smaller gifts are, however, naturally recorded in the annual reports, with the name of the soldier in commemoration of whom they were subscribed, and in view of the part which the hospitals are playing in the treatment of the wounded, it seems that many people who cannot afford to endow or to name a bed, might desire and be encouraged to give smaller sums as memorial gifts of this character. We throw out the suggestion for what it may be worth, merely recording the encouraging fact that one or two Scotch hospitals have already benefited by it.

### AN INDEFENSIBLE PRACTICE.

To disguise the annual report is one method of making it attractive. As an instance we may quote the "short account" of the Orphans' Home, Austral Street, West Square, S.E., compiled by Miss Sharman rather in the form of a chatty narrative than in that of a business record. The work of the Home is divided into several branches, and as a way of impressing a list of them upon the memory it will be interesting to future generations to learn that "twice the Zeppelins have been over the London Home, several times over the Gravesend Home, and once the raiders passed over four of our Homes, visiting London, Gravesend, Stockwell and Tunbridge Wells." A feature of the institution's resources appears to be the large number of gifts in kind that are received. In the London Home are nearly two hundred children, many of whom are trained for domestic service. Some of the branch Homes have been temporarily closed, whereby, the report laments, the number of children has been decreased from 365 to 324. The following passage is worth quoting in this connection: "Through the kind gift of the Ogilvie Charity, some of our dormitories have been supplied with single instead of double beds, which, though it is an improvement in many ways, has somewhat decreased our sleeping accommodation." If this means that it has been the custom for more than one child to be placed in a bed—then the diminution in the number of children in the Homes is a matter for congratulation. To put more than one child in a bed is an indefensible practice, and cannot be tolerated with any pretensions to high standard or up-to-date administration. Two children in one bed should lead to the withdrawal of many subscribers.

### THE ADDINGTON PARK WAR HOSPITAL.

An institution of a very specialised kind has been organised for over a year under the above title; and many very interesting details about it have recently been made public, with the consent of the Director-General, Army Medical Service, by Dr. E. C. Hort, honorary physician and director of the laboratory. Originally conceived as a hospital for acute infective disorders, such as enteric, dysentery, septicæmia, it was launched fully equipped at the expense of private subscribers and of the Red Cross; the entire cost of maintenance is borne by the War Office, and an officer of the A.M.S. (Col. Barrow) is Commandant. Very soon it was found advisable to change the scope of the hospital's work, and to make it a clearing house for "carriers" and for the investigation of cases invalided from our forces abroad with a view to detecting carriers. Hence a large proportion of the patients are in a convalescent condition, awaiting the necessary bacteriological inquisition before being allowed to go on furlough or to convalescent homes. Successive expansions have been necessary, and the hospital now provides (or soon will do so) accommodation for

1,700 patients. Dr. Hort's paper is largely technical, dealing with the methods in use for isolating and identifying the numerous organisms which may occur in the stools and urine of patients convalescing from the enteric and similar fevers. The results of this work, which occupies the time of a staff of five bacteriologists, are promised for publication at the end of the war, or possibly sooner: almost the only hint dropped of impressions gained or conclusions reached is to the effect that the anti-typhoid inoculation tends to reduce the proportion of carriers among convalescents from that disease. Evidently this very specialised hospital plays a most important rôle in the prevention of epidemic disease, and renders valuable service not merely to the Army but to the whole community.

### EXPERIENCES OF A CAPTURED OFFICER OF THE R.A.M.C.

In the last issue of the *Journal of the Royal Army Medical Corps* one of the medical officers captured at the battle of Le Cateau in August 1914, and detained by the Germans for ten months in flagrant violation of the Geneva Convention, records his experiences as a prisoner of war in several different camps. These were much on a par with those of other officers, and confirm the shocking accounts of German inhumanity which are all too familiar in this country already. On the actual field of battle this officer was well treated, but after that a tale of ill-treatment begins, varied only by rare exceptions. The official excuse for his detention was that he was not being kept as a prisoner of war, but to look after the many thousands of British wounded whom the Germans expected to take at some future date. This is, of course, the stock official attitude whenever protests about the violation of the Geneva Convention are handed in by imprisoned medical officers. On inquiry why, in that case, medical officers are kept in close detention in the prison camps, the official answer is that it is to protect them from the fury of the populace. For five months Captain Beaman had no medical work at all; then he was sent to a camp where seventy British wounded were interned, nearly all of whom had recovered. After five months more he was released. His account of the conditions in which the unfortunate rank and file have to live is simply heartrending. The officers are treated badly enough; but the German object is apparently to let starvation, overcrowding, harsh usage, and preventible disease work the greatest possible havoc on the prisoners in these camps for the rank and file. Tuberculosis is terribly prevalent, and nothing is done to check its ravages.

### THE WESTERN HOSPITAL, TORQUAY.

The annual report of this hospital discloses much excellent work and a liberal response by friends of the institution to the cost of providing treatment for wounded soldiers. The number of beds was increased from forty to seventy-two, not including eighteen for which room was found in a house close to the hospital provided and equipped by the generosity of two anonymous donors. The whole of these ninety beds are at the disposal of the military authorities: all but two of the subscribers agreed that their money should be made use of in this way instead of for the usual charitable purposes of the hospital. Not only so: an $x$-ray apparatus has been installed and an operating theatre built, both provided by anonymous donors, and the increased nursing staff are being housed by yet another friend. Sir Rudolph Hampden Smith has undertaken the duties of medical superintendent, and other retired members of the visiting staff have resumed duty. The War Office pays 3s. 6d. per day for each patient, and the rest of the cost of maintenance comes from subscriptions and interest on investments. As the result of fifteen months' working a debit balance of £262 has been replaced by a credit of £28, a most excellent result. The only point in the accounts which we cannot understand is how the dividends for eighteen months on funds of nearly £11,000, invested in gilt-edged securities yielding on the average nearly 4 per cent., amount to but £279, including refund of income tax. As the accounts are audited, there is doubtless some simple explanation, but we are puzzled to know what it can be.

### THE INDIAN ARMY CORPS HOSPITALS.

The withdrawal of the Indian troops from France for service in other parts of the world-wide war began some months ago, and is now complete we believe. This redistribution of forces involves, naturally, the removal of many hospitals and ambulances both from France and from Great Britain and their transfer to other spheres of duty. Thus several buildings are released from occupation as hospitals for Indians and have become available for similar usage for our own troops. It is stated that the only accommodation retained in this country consists of the Brighton Pavilion, the Lady Hardinge Hospital at Brockenhurst, and the convalescent depôt at Barton, in Hampshire. Presumably these, too, will be evacuated in a few weeks or months, when the occupants are sufficiently convalescent to be transferred to India or to return to their regiments. The occasion has been taken by the Chief Constable of Brighton, where many of the Indian sick and wounded have been treated, to publish a very gratifying testimonial to the uniform good conduct of the Indian soldiers, who seem to have upheld the honour of their races as manfully in England as in France.

### THE LACK OF ISOLATION HOSPITALS IN CORNWALL.

How inadequately provided with isolation hospitals is the county of Cornwall is apparent from the annual report of the new medical officer, Dr. E. M. Clarke, who was appointed last year in the place of Dr. Burnet. Schemes for establishing isolation accommodation in combined areas were prepared by the late medical officer, but so far the various authorities concerned have been deterred by the expense from taking action, and at the present time the financial outlook is not encouraging. On the other hand, the presence of troops in camps and billets in many parts of the county has emphasised the need for more provision for isolating cases of infectious disease. Those acquainted with the rural districts of Cornwall will not be surprised that the improvement in sanitary administration makes a somewhat sluggish progress in the county, but there is hope that the energy of the new C.M.O. will bear fruit, and that, in the provision of isolation hospitals at any rate, the district councils will meet the pressing demands.

### FIRST-AID FOR STREET ACCIDENTS.

Some enterprising tradesmen in Battersea have combined to provide a set of apparatus for first-aid requirements for use in accidents and emergencies occasioned by the darkened streets. On a wall opposite a draper's shop in Wandsworth Road, Battersea, is fixed a small case containing splints, bandages, and dressings. A notice informs those whom it may concern that a key of the case is kept in the draper's shop; but in case the contents are required when the shop is closed, another key can be obtained by smashing a glass panel in the front of the case itself. The whole idea is eminently praiseworthy, though the temptation to irresponsible individuals to purloin the contents of the case may, we fear, prove irresistible. There is no doubt that accidents are favoured by the darkness of the streets, and London could do with more safeguards such as that provided by the thoughtful Battersea tradespeople.

### COAL SUPPLIES AT INFIRMARIES.

There are still many institutions where the supply of coal is far from satisfactory, but where these cases have arisen they are frequently due to unbusinesslike methods adopted by those in authority. At the Bermondsey Infirmary the supply of coal was so low just before Christmas that there was a grave fear of a famine, which would have resulted in great sufferings to the patients. But the cause was due entirely to the Board of Guardians. Early last year they entered into a contract for a specific kind of coal at 21s. per ton, but, as the contractors held that they were unable to supply this particular coal, they were permitted to supply another coal at 24s. a ton. There was a lapse of some weeks ere the Guardians found out what had been done, as the alteration was carried out by the responsible officers at a time when the board was in recess. The Guardians at once stopped the supply of coal at 24s. a ton, and called upon the contractors to give them the coal originally contracted for. This they were unable to do, and the board decided to cancel the contracts, and to advertise for fresh tenders. Only one was received at a greatly enhanced price, and so the board had to go to the old contractors asking for a supply of the coal at 24s. a ton. But the contractors found that the price of this coal had gone up, and so the Guardians had to agree to have a supply of this particular coal at the greatly increased price of 32s. a ton. They were told that under the circumstances they were lucky to get any coal at all. One cannot be surprised at the result, considering the way the Guardians have treated their contractors.

### THE LATE SIR FREDERIC HEWITT.

The not unexpected but regretful death on January 6 of Sir F. W. Hewitt, M.D., M.V.O., after an illness of some months' duration, removes one who was long recognised as the premier practitioner of anæsthetics in London, and probably in the world. Sir Frederic was known far and wide as anæsthetist to the King and to the late King Edward; and had a long and honourable record of institutional service. Originally educated at Cambridge and St. George's, he became in the 'eighties anæsthetist to Charing Cross Hospital, to the Royal Dental Hospital, and to the Waterloo Hospital for Women and Children. His best known institutional connection, however, was at the London Hospital, where he was associated with Sir Frederic Treves. At one time in the early 'nineties Hewitt was giving over 3,000 anæsthetics a year in hospitals alone; and he was a man who never spared himself. Of an ingenious and mechanical turn of mind, he devised very many new apparatus for use in anæsthetics and improved many old ones. Of all his inventions, perhaps that for the administration of gas and oxygen will live the longest; but there was scarcely a tool in the anæsthetist's bag in which he did not bring about improvements. His textbook of anæsthetics, now in a fourth edition, has been recognised for years as the standard work on this subject in the English language. When in 1901 he anæsthetised the late sovereign for the operation which Sir F. Treves performed, it was felt by the institutional and medical worlds that the M.V.O. was a somewhat shabby recompense for the assumption of a responsibility many times greater than that of the operator. Indeed, history may testify the truth of this, and record how King Edward's tactful sympathy saved the situation. It can safely be asserted that King Edward must have been a very bad subject for anæsthesia, and that the risk of anæsthetising him in all the circumstances was not small. Several years later Hewitt was knighted. He was in his fifty-ninth year at the time of his death. We knew him as a most kindly, thoughtful, helpful colleague who always put the patient's interests first.

### THE GREEN CROSS.

The Red Cross for men, and the Blue Cross for animals, are already familiar, but the Green Cross has, at first, something unusual about it, like the green carnation. It is, however, the badge of the Women's Reserve Ambulance (Green Cross Society), whose headquarters are at 199 Piccadilly. The W.R.A. meet trains at the railway stations and convey soldiers, newly arrived on leave, to the Y.M.C.A. huts and other stations in cars supplied by the organisation, whose transport section also carries materials for the Munitions Committee. The membership of the Women's

Reserve Ambulance now numbers 300, some of whom hope for an opportunity of foreign service when sufficient money has been subscribed to supply the motor field operating theatre which is understood to have been asked for by the French Red Cross. The uniform is of khaki, with a slouch hat, and a green Maltese cross.

### ENDOWED BEDS AT CARDIFF HOSPITAL.

No fewer than four beds have been endowed at King Edward VII.'s Hospital within the past month, cheques amounting to 1,000 guineas each having been sent to the hospital by the following well-known firms at Cardiff Docks:—Messrs. Evans & Reid, Ltd., Messrs. Morgan & Cadogan, Ltd., and Messrs. Watts, Watts, & Co., Ltd. The fourth bed was endowed by a similar gift from Captain David T. Lewis, R.A.M.C., as a tribute to the memory of his father, the late Alderman Richard Lewis, vice-chairman of the Glamorgan County Council. Counting three other beds endowed some months ago—two by Alderman W. H. Mathias, of Porth, and one by Sir William James Thomas (in memory of the late Nurse Cavell)—seven beds have been endowed at this hospital within the past twelve months. It may also be recalled that Colonel Bruce Vaughan only a few months ago handed over £5,000 to the hospital, the gift of an anonymous donor for the building of a maternity flat. The hospital is to be congratulated on these generous gifts which may fairly be regarded as a recognition of its good work on behalf of wounded soldiers and for the civil population. These gifts are an earnest that a hospital which has stuck manfully to its duty will not be permitted to go unrewarded, especially when it has one of the ablest chairmen and secretaries in the country.

### THE RECREATION ROOM OF THE FUTURE.

THE want of recreation rooms in those hospitals which are treating wounded soldiers has been generally felt, and it is pleasant to record the special huts which have been given or built to supply this need at Lincoln, Norwich, and other hospital centres. In spite of convalescent homes attached to hospitals many of the wounded are in a semi-convalescent state, and those hospitals which are situated in the larger county towns have felt the need of providing some means of recreation outside the wards, and at the same time independent of the town itself, which has usually been placed out of bounds for the convalescents. But the function and value of the recreation hut does not end here. Apart from its normal use for writing, reading, and quieter games, it provides a means of holding the periodic entertainments which are now a normal feature of hospital life, with a staff of volunteers to organise and to conduct them. It is interesting to speculate on the use to which these huts will be put after the war. In mental hospitals their equivalent has long been a feature. Can we doubt that the departure of the wounded will leave a legacy of more active entertainment for hospital patients than was accorded them in the past?

### A NEW MEDICAL PLAYWRIGHT.

BY the time these lines appear in print the public will have had an opportunity of seeing on the London stage a new play by an author who is also a medical practitioner, though already known as a novelist who has made provincial practice the subject of one of his works. Mr. Frank G. Layton, the new playwright, introduced by Miss Horniman to the Duke of York's Theatre this week with a play entitled "The Parish Pump," deserves mention and encouragement as one of the few writers who are also medical men that deal with their own profession in their books. A great service both to art and to the public would be paid by the representation in a novel or on the stage of hospital life and medical practice as they really are. Yet our doctors who are writers seem to fight shy of this virgin field which, of all authors, they must know best. Mr. F. G. Layton, M.R.C.S., whose novel, "Doctor Grey," was known to many under the pseudonym of Stephen Andrew, has, however, once at least essayed the making of a play out of the materials provided by the consulting room. The play in question, called "Philip's Wife," which was published by A. C. Fifield in 1914, was reviewed in THE HOSPITAL on its appearance. Its characters include two medical portraits, and it deals with one of the dilemmas which professional secrecy imposes on the doctor. We hope that Mr. Layton will again turn to his profession, or memories of hospital life, in a future drama.

### THE ORGANISATION OF WOMEN SUBSCRIBERS.

Now that so many men have left the workshop for the camp, it has become necessary to see what can be done to make good the loss of subscriptions involved in their departure. In many instances, as we all know, women have taken the place of men as wage-earners, but it is doubtful if adequate efforts have been made to persuade them of their duty to follow the example of the men in making a regular weekly contribution to the funds of the local hospital. It is true that women are, to say the least, not less generous than men, indeed they are often more so, but at the same time they may be encouraged to form the habit of weekly giving which has been such a regular feature in the lives of the men whose place they are taking to-day. Whether or not a man secretary is the best person to seek their sympathies may be open to question, but at all events it is well worth while to pay special attention at this juncture to the organisation of women subscribers in every district where they are replacing men in workshop or factory. Under existing circumstances their special duty to the hospitals is obvious. It is necessary to point it out if a new class of subscribers is to be enrolled.

### PRAISE FOR THE STRETCHER-BEARERS.

SIR IAN HAMILTON, in his tragic but historic dispatch, conveyed a well-merited tribute to the work of the stretcher-bearers at Gallipoli. "A

feature of every report, narrative, or diary I have read,'' he writes, ''has been a tribute to the stretcher-bearers. All ranks, from Generals in command to wounded men in hospital, are unanimous in their praise. I have watched a party from the moment when the telephone summoned them from their dug-outs to the time when they returned with their wounded. To see them run light-heartedly across fire-swept slopes it is to be privileged to witness a superb example of the hero in man. No braver corps exists, and I believe the reason to be that all thought of self is instinctively flung aside when the saving of others is the motive.'' Sir Ian's words refer, doubtless, both to the bearers of the Royal Army Medical Corps units and to the ''regimental stretcher-bearers,'' who are combatant soldiers detailed for stretcher duties. His praise confirms the testimony of those who have seen the work of the bearers in France, where the majority of the opportunities have fallen to these carriers of rescue.

### HONOUR FOR EX-HOSPITAL CHAIRMAN.

The board of management of the Blackburn and East Lancashire Royal Infirmary have recorded their appreciation of the honour lately conferred on Sir John Rutherford, Bart., M.P., who has been an active office-holder of the institution since he first joined the board in 1889. Now a trustee and vice-president, Sir John formerly occupied the position of chairman for seven years. In 1888-89 he was Mayor of Blackburn, and his interest in institutional affairs and the voluntary system appears from the fact that he is also governor of the Cotton Districts' Convalescent Fund. Although Sir John Rutherford has given much of his time to politics, and has been in the House of Commons for twenty years, he has had other interests, and for an even longer period has been actively associated with the hospital.

### A COSTLY ALTERATION.

The Southwark Board of Guardians have been presented with a bill for £5,032 by the Lambeth Board of Guardians, with a request that they will send it on to the War Council for the latter to issue a cheque for the amount. The sum is the cost of converting a part of Renfrew Road Workhouse into a temporary infirmary to provide accommodation for patients from Southwark, who were removed when the infirmary was taken over as a military hospital. Some 250 Southwark patients have found accommodation at Lambeth, and the amount involved includes such items as £600 for an electric lift, and other expenditure that should not have been undertaken for a temporary infirmary, which may only be used for a few months. The Inspector of the Local Government Board had already cut down some of the suggestions of the Lambeth Guardians, but the account as it now stands represents in the bulk the amount expended on furnishing. The Southwark Board of Guardians have declined absolutely to pass the account, as they consider that the greater part of the money has been expended on luxuries which were unnecessary for a temporary infirmary. Legally under their agreement with the Lambeth Board they are bound to send the account on to the War Council for them to meet; but in view of the extortionate demands and the further fact that they were in no way consulted, they feel they have a deep grievance in what they think is a wanton waste of public money at the present time.

### A GRAND OLD YACHT.

Lord Brassey has placed at the disposal of the Government of India his steam-yacht *Sunbeam*, which was also utilised for the same purpose last autumn in the Mediterranean. There can be very few yachts in commission which have seen as much active service as the *Sunbeam*, for it must be more than forty years since the first Lady Brassey made the ship a household word by her account of a voyage round the world in it. A few years ago, it will be remembered, the gallant old yacht was entered for a Transatlantic race for sailing yachts, and made quite a good showing among the younger and larger boats, though not the first to reach the goal. We trust the Red Cross will prove a protection against the torpedoes of the pirates, for the *Sunbeam* is in many ways a historic craft.

### THE BRITISH WOMEN'S HOSPITAL.

A hospital unit bearing this name is to start for Russia very shortly, and to devote its energies to maternity work among the vast population of refugees from the war zone who have crowded into Petrograd. The unit is being sent out fully equipped and staffed by the National Union of Women's Suffrage Societies, and a building which is to be finished shortly will be handed over to the unit on its arrival in the Russian capital. The Tatiana Committee, it is also stated, is to make a contribution towards equipment and upkeep; this committee has already done great work in the provision of shelter, fuel, and food for the Polish and Russian refugees. The Empress Alexandra has granted the Women's Hospital her protection, while the Grand Duchess Kyril and Lady Georgina Buchanan are its immediate patronesses. The total cost for six months' work is estimated at £5,000.

### AN ACTIVE SERVICE EXHIBITION.

On behalf of the British Red Cross Society and the Order of St. John of Jerusalem an ''Active Service Exhibition'' is being arranged at the Prince's Skating Hall, Knightsbridge, from March 18 to April 8. There will be realistic representations, it is announced, of the actual conditions under which our soldiers and sailors fight and live; inasmuch as the designs and execution of them are to be entrusted to experts from the seats of war, it is just possible the public may see and appreciate what life in the trenches really is like, though there are many horrors there (apart from German shelling and sniping) too grim for reproduction anywhere. The work of the Red Cross and of the Order will also be comprehensively represented, and a large collection of war relics of all kinds will be brought together. Further particulars may be had on application to Room 102, 83 Pall Mall, S.W., the headquarters of the London branch of the British Red Cross Society.

### THE SERBIAN RETREAT.

A very vivid and graphic account of the adventures of a London medical student (Mr. Vincent Drew, of Guy's Hospital) during the retreat of the Serbians before the Bulgarian and Austro-German armies has appeared in the *Times*. Mr. Drew went to Serbia last March as dresser to the First British Field Hospital, and at the beginning of October was at Pirot. As the communications were even then being threatened he was instructed to proceed *via* Nish to Krvshevatz. Eventually, through blizzards of snow, he reached Prishtina, and thence Prizrend. Here he found himself at the end of his resources, and thought of abandoning further retreat and of waiting to be taken by the Austrians; but an American doctor lent him some money, so Mr. Drew crossed the border into Montenegro. Over icy passes, and in a never-ending stream of refugees of all nations, the flight continued. Fortunately he was able to hire a couple of horses, and eventually reached Podgoritza, whence he travelled on to Cettinje and Skutari, to report to Sir Ralph Paget. Last of all he tramped to Durazzo, whence he sailed for Brindisi and from there home.

### PUBLIC HEALTH AND THE PRICE OF LAND.

The presence of a Medical Officer of Health at a conference on such public questions as that of housing usually adds a practical, as well as a scientific, value to the suggestions, and this was certainly the case at the first Scottish Conference on Housing, held at Glasgow last week. Dr. Chalmers, Medical Officer of Health for the city, for instance, in dealing with the price of land in cities, pointed out that the quickest way to reduce the price of land in these populous areas was to reduce the height of tenement houses. The time had come, he remarked, for the abolition of the apartment houses. His suggestion for reducing the height of tenement houses is interesting in more ways than one, for while the commercial aspect, with which Dr. Chalmers was dealing, has an important bearing on the public health, the general modern tendency to deprive our streets of sunlight by building shops and houses considerably higher than the width of the streets warrants is an evil of grave importance to the population. The evil of the tenement house is one which can hardly be attacked from too many sides, and the Medical Officer of Health has the advantage of his experience in appreciating its commercial, as well as its sanitary, aspects.

### THE PRIVATE PATIENT IN NEW ZEALAND.

At a special meeting of the Otago Hospital and Charitable Aid Board, the New Zealand Minister of Public Health had some interesting remarks to make on the provision of special wards for the richer class of paying patient in New Zealand hospitals. He was asked to discuss the question whether a hospital board had power to give private treatment at special fees above the ordinary rate to a patient seeking admission. The Minister, in reply, stated that in connection with the mental hospitals an application had been made to him by a gentleman willing to pay £6 a week if his wife, a mental case, might receive the refinement of her home surroundings in hospital. The Minister's opinion was that anything outside the ordinary treatment at the ordinary rate was impracticable in existing institutions, but he was prepared to support a scheme for the erection of separate buildings, contiguous to the institutions and under the control of their medical staffs, where such cases might be received on special terms. It is encouraging to have the New Zealand Minister of Public Health advocating in principle the system of graduated pay pavilions, so that every class from the poor to the wealthy may be suitably provided for.

### ANTI-TOXIN AND THE FIRE BRIGADE.

The local authority of Edmonton has conceived a scheme for making use of the fire brigade depôt in connection with the anti-toxin treatment. The proposal which has been brought forward is for the keeping of syringes and serum at the brigade station so that when medical practitioners find they have a case of diphtheria they can without delay administer anti-toxin treatment. This scheme has found much favour, though the local authority is holding it up for the moment pending a decision as to the payment of a fee of 3s. 6d. to a medical man for administering the serum. In other boroughs it has been the practice to appoint three or four leading chemists in different parts of the district to hold and supply such a stock of serum for use in necessitous cases: this is the first time that we have heard of a fire station being used as a depôt in connection with such a scheme.

### THIS WEEK'S DRUG MARKET.

The advancing tendency in the prices of drugs continues, and so far as can be foreseen most of those medicinal products which have been increasing in value ever since the outbreak of war will move in the same upward direction so long as the war lasts. The promised increase in the output of salicylates and other drugs has been so long deferred that buyers have almost ceased to expect that the promise will ever be fulfilled, and there are certainly at present no signs that the position will be relieved in the near future. Salicylates, as a matter of fact, are dearer than they were at the end of the year, and the same applies to some of the other commonly prescribed drugs. The extremely high values of bromides are well maintained. Hypophosphites are dearer. Makers of caffeine have raised their nominal quotations, which are still, however, very much below the prices that can be obtained by those who hold small stocks of caffeine. English makers of quinine have also raised their quotations, but here again makers' prices are much below those that are being paid for Continental brands of quinine. Cocaine is rather dearer. There has been no further change in the position of ipecacuanha, but prices are well maintained. Paraldehyde is dearer, as also is strychnine.

